# miR-4317 suppresses non-small cell lung cancer (NSCLC) by targeting fibroblast growth factor 9 (FGF9) and cyclin D2 (CCND2)

**DOI:** 10.1186/s13046-018-0882-4

**Published:** 2018-09-18

**Authors:** Xi He, Si-yuan Chen, Zhao Yang, Jie Zhang, Wei Wang, Mei-yue Liu, Yi Niu, Xiao-mei Wei, Hong-min Li, Wan-ning Hu, Guo-gui Sun

**Affiliations:** 1 Department of Thoracic Surgery, North China University of Science and Technology Affiliated People’s Hospital, Tangshan, 063000 China; 2 Department of Radiation Oncology, North China University of Science and Technology Affiliated People’s Hospital, Tangshan, 063000 China; 3 Department of Pathology, North China University of Science and Technology Affiliated People’s Hospital, Tangshan, 063000 China

**Keywords:** Non-small cell lung cancer, Metastasis, Prognostic biomarker, Therapeutic target

## Abstract

**Background:**

Non-small cell lung cancer (NSCLC) is a leading cause of death worldwide. MicroRNAs (miRNAs) have been indicated as crucial actors in cancer biology. Accumulating evidence suggests that miRNAs can be used as diagnostic and prognostic markers for NSCLC.

**Methods:**

The purpose of this study was to characterize and identify the novel biomarker miR-4317 and its targets in NSCLC. The expression of miR-4317 was analyzed by in situ hybridization (ISH) and quantitative reverse transcription polymerase chain reaction (qRT-PCR). The effect of miR-4317 on proliferation was evaluated through 3–4,5-dimethylthiazol-2-yl-5-3–carboxymethoxyphenyl-2-4-sulfophenyl-2H-tetrazolium (MTS) and colony formation assays, and cell migration and invasion were evaluated through transwell assays. The expression of target proteins and downstream molecules was analyzed by qRT-PCR and western blot. Dual-luciferase reporter assay was used to assess the target genes of miR4317 in NSCLC cells.

**Results:**

Our results demonstrated that miR-4317 was downregulated in NSCLC tissues and serum, particularly in lymph node metastasis and advanced clinical stage tissues. Kaplan-Meier survival analysis showed that NSCLC patients with high expression of miR-4317 exhibited better overall survival (OS). Enhanced expression of miR-4317 significantly inhibited proliferation, colony formation, migration and invasion, and hampered cycles of NSCLC cell lines in vitro. Our results suggested that miR-4317 functions by directly targeting fibroblast growth factor 9 (FGF9) and cyclin D2 (CCND2). In concordance with in vitro studies, mouse xenograft, lung, and brain metastatic studies validated that miR-4317 functions as a potent suppressor miRNA of NSCLC in vivo. Systemically delivered agomiR-4317 reduced tumor growth and inhibited FGF9 and CCND2 protein expression. Reintroduction of FGF9 and CCND2 attenuated miR-4317-mediated suppression of migration and invasion in NSCLC.

**Conclusions:**

Our results indicate that miR-4317 can reduce NSCLC cell growth and metastasis by targeting FGF9 and CCND2. These findings provide new evidence of miR-4317 as a potential non-invasive biomarker and therapeutic target for NSCLC.

**Electronic supplementary material:**

The online version of this article (10.1186/s13046-018-0882-4) contains supplementary material, which is available to authorized users.

## Background

MicroRNAs (miRNA) are a class of small noncoding RNAs of 18–24 nucleotides that bind to partially complementary recognition sequences of mRNA, causing either degradation or inhibition of translation and thus effectively silencing their mRNA target [1]. To date, more than 1881 human miRNAs have been registered in miRBase (Release 21; http://www.mirbase.org/). Massively parallel signature sequencing of miRNAs can identify the miRNome in-depth, revealing differences in miRNA expression as well as individual miRNA abundances. Recent evidence suggests that dysregulation of miRNAs is involved in several biological processes, such as development, differentiation, proliferation, and apoptosis [2, 3]. miRNAs have also been suggested to play essential roles in initiation and progression of certain types of cancer, such as lung cancer [4, 5], breast cancer [6], and colorectal cancer [7]. Although classical approaches to cloning have partially revealed miRNA expression profiles in a panel of mammalian tissues and cell types [8, 9], low throughput, low sensitivity, and poor resolution make these approaches unlikely to define the miRNome.

Lung cancer is one of the most frequently diagnosed cancers and the leading cause of cancer-associated death both in men and women worldwide [10]. It was estimated that there were 1.8 million new cases in 2012, leading to the death of approximately 1.59 million people per year globally, extrapolating from a 2012 International Agency for Research on Cancer risk assessment [11], and this trend is expected to continue until 2030. Approximately 85% of lung cancers are classified histopathologically as non-small cell lung carcinomas (NSCLCs) [12]. Although treatment advances have been achieved with the use of surgery and chemotherapy [13], and targeted therapies (EGFR or ALK tyrosine kinase inhibitors) have significantly improved the outcomes of some subgroups of advanced NSCLC patients, the 5-year overall survival (OS) rate is only 16% for all stages of the disease [14]. The poor outcomes and frequent relapses associated with lung cancer urgently demand the development of new screening methods and early biomarkers for accurate and non-invasive detection of lung cancer metastasis and recurrence [15, 16]. Therefore, there is a great need for the development of strategies for improved diagnosis, prevention, and therapy for NSCLC.

Several studies have shown that miRNAs can be used as diagnostic and prognostic biomarkers [4–7, 17–23]. However, the majority of previous studies have been based on small sample sizes, relatively limited numbers of miRNAs, or limited miRNA detection methods [5, 19–23]. In NSCLC, several miRNAs are essential for tumor development, including miR-9, let-7, and miR-193a-3p [24–26]. Downregulation of miRNAs, such as miR-10b, − 143, −181a, − 21, − 205, − 221, − 222, and -449a, has been shown to be a key factor in NSCLC tumorigenesis [27]. However, the expressions of other miRNAs, including miR-106a, − 146, − 155, − 150, − 17-3p, − 191, − 197, − 192, − 21, − 203, − 205, − 210, − 212, and − 214, have been reported to be upregulated in lung cancer [5]. Our previous study of 32 lung adenocarcinoma patients with brain metastasis (BM) versus 55 patients without BM using miRNA array profiles demonstrated that miR-4317 expression levels can predict OS in NSCLC. However, the precise molecular mechanism through which miR-4317 influences NSCLC progression remains largely unknown. In this study, we identified a new miRNA and signaling pathway for understanding the pathogenesis of NSCLC, providing a promising biomarker and therapeutic target for NSCLC.

## Methods

### Human NSCLC tissue samples

A chip including 162 cases of non-metastatic NSCLC tissues and non-neoplastic lung tissues was purchased from Outdo Biotech (HlugA180Su02, Shanghai, China; http:// www.superchip.com.cn/). Another 140 paired NSCLC tissues and matched adjacent non-cancerous tissues were collected in North China University of Science and Technology Affiliated People’s Hospital from 2012 to 2015. Histologic characteristics of these samples are summarized in Table 1. Serum samples from 86 NSCLC patients and 40 healthy controls were obtained from the abovementioned hospital (Table 2). All serum specimens were transported at 4 °C and stored at − 80 °C until RNA extraction. None of the patients had received preoperative treatments such as radiotherapy or chemotherapy. The methods used to treat the tissues were carried out strictly in accordance with institutional policies and approved guidelines for experiment procedures.

**Table 1:** Correlations between miR-4317 expression and clinicopathological features in patients with NSCLC

Feature	Training group (*n* = 162)			Test group (*n* = 140)		
	Low (*n* = 120)	High (*n* = 42)	*p*	Low (*n* = 102)	High (*n* = 38)	*p*
Gender						
Male	89 (74.2)	28 (66.7)	0.35	65 (63.7)	26 (68.4)	0.60
Female	31 (25.8)	14 (23.3)		37 (36.3)	12 (31.6)	
Age^a^						
< 60 y	45 (37.8)	15 (35.7)	0.81	43 (42.2)	16 (42.1)	1.00
≥ 60 y	74 (62.2)	27 (64.3)		59 (57.8)	22 (57.9)	
Tumor size^a^						
< 5 cm	74 (31.7)	29 (48.5)	0.39	69 (69.0)	28 (73.7)	0.59
≥ 5 cm	46 (68.3)	13 (51.5)		31 (31.0)	10 (26.3)	
Tumor stage						
T1 + T2	90 (75.0)	35 (83.3)	0.27	79 (77.5)	33 (86.8)	0.22
T3 + T4	30 (25.0)	7 (16.7)		23 (22.5)	5 (13.2)	
Histological type						
Adenocarcinoma	63 (52.5)	24 (57.1)	0.60	59 (57.8)	17 (44.7)	0.17
Squamous cell carcinoma	57 (47.5)	18 (41.9)		43 (42.2)	21 (55.3)	
Histological grade^a^						
Well/moderate	88 (73.3)	27 (64.3)	0.27	57 (61.3)	30 (78.9)	0.05
Poor/NS	32 (26.7)	15 (35.7)		36 (38.7)	8 (21.1)	
Lymph node metastasis^a^						
Negative	49 (51.6)	32 (80.0)	0.002	58 (59.8)	33 (86.8)	0.003
Positive	46 (48.4)	8 (20.0)		39 (40.2)	5 (18.5)	
TNM stage^a^						
I + II	58 (49.6)	36 (87.8)	0.000	61 (60.4)	34 (89.5)	0.001
III + IV	59 (50.4)	5 (12.2)		40 (39.6)	4 (10.5)	

Data are presented as n (%)

^a^Sum does not equal the total number due to missing data

**Table 2:** Correlations between serum expression of miR-4317 and clinicopathological features in 86 patients with NSCLC

Feature	Serum expression of miR-4317		
	Low (*n* = 43)	High (*n* = 43)	*p*
Gender			
Male	27 (62.8)	29 (67.4)	0.65
Female	16 (37.2)	14 (22.6)	
Age			
< 60 y	17 (65.4)	19 (44.2)	0.66
≥ 60 y	26 (34.6)	24 (55.8)	
Tumor size			
< 5 cm	30 (69.8)	33 (76.7)	0.47
≥ 5 cm	13 (30.2)	10 (23.3)	
Tumor stage			
T1 + T2	31 (72.1)	38 (88.4)	0.06
T3 + T4	12 (27.9)	5 (11.6)	
Histological type			
Adenocarcinoma	30 (69.8)	23 (53.5)	0.12
Squamous cell carcinoma	13 (30.2)	20 (46.5)	
Histological grade^a^			
Well/moderate	30 (71.4)	28 (65.1)	0.53
Poor/NS	12 (28.6)	15 (34.9)	
Lymph node metastasis			
Negative	18 (41.9)	34 (79.1)	< 0.0001
Positive	25 (58.1)	9 (21.9)	
Clinical stages			
I + II	20 (46.5)	33 (76.7)	0.004
III + IV	23 (54.5)	10 (23.3)	

Data are presented as n (%)

^a^Sum does not equal the total number due to missing data

### MicroRNA microarray assay

The analysis of miRNA microarray data was conducted in 32 clinical samples acquired from lung adenocarcinoma patients with BM in contrast to 55 patients without BM obtained from the Cancer Hospital, Chinese Academy of Medical Sciences (Beijing, China) between 2003 and 2008. In short, total RNA isolated from patient samples was examined with the mammalian miRNA array V2.0 (CapitalBio, Beijing, China), which identifies 1105 miRNAs in humans, mice, and rats. Separation of low-molecular-weight RNAs from total RNA was carried out by a PEG precipitation method; the low-molecular-weight RNAs were then labeled with 5-phosphate-cytidyl-uridyl-Cy3–3 and then hybridized to the mammalian miRNA array overnight at 42 °C. A LuxScan 10 K/A laser confocal scanner was used to scan the arrays, and the acquired images were evaluated using LuxScan 3.0 software (both from CapitalBio). Cluster 3.0 was used to carry out clustering analysis, and the results were viewed with TreeView software. The normalization of fluorescence signals was carried out using the median center tool for genes in Cluster 3.0, and they were evaluated using the significance analysis of microarrays (SAM), with a false discovery rate (FDR) threshold set of 0 and fold-change established at ≥2- or ≤ 0.5-fold change and *p* value < 0.05.

### Cell lines and cell culture

All NSCLC cell lines used in this study, including A549, NCI-H1299, NCI-H157, ANIP-973, GLC-82, and NCI-H292, were cultured in 1640 RPMI medium supplemented with 10% fetal bovine serum at 37 °C in a humidified atmosphere containing 5% CO_2_. The human fetal lung fibroblast cell line (MRC-5) was cultured in Minimum Essential Medium (MEM) containing non-essential amino acids, Earle’s salts, and L-glutamine supplemented with 10% fetal bovine serum and 1% antibiotic-antimycotic solution (containing 100 U/mL penicillin, 100 μg/mL streptomycin, and 0.25 μg amphotericin), and was maintained in a humidified air atmosphere with 5% CO_2_ at 37 °C.

### In situ hybridization (ISH) of miR-4317

ISH was performed per the manufacturer’s instructions. The miR-4317 probe was tagged with 3′ and 5′ digoxigenin and LNA modified (Redlandbio.biomart.cn, Guangzhou, China). The probe-target complex was detected using an antidigoxigenin-alkaline phosphate conjugate and nitro-blue tetrazolium and 5-bromo-4-chloro-3′-indolyphosphate as the chromogen. Cases were classified according to the cytoplasmic miR-4317 intensity as follows: negative = negative or faint expression in most cells; low expression = low expression in most cells or moderate expression in < 50% of the cells; high expression = moderate to strong expression in most cells.

### miRNA transfection

All endogenous mature miRNA mimics, inhibitors, and agomirs were purchased from RiboBio (Guangzhou, China). For transfection, experimental protocols were performed according to the manufacturer’s instructions (RiboBio). The miRNA mimics, miRNA inhibitors, and miRNA NC were transfected into cells using Lipofectamine 2000 (Invitrogen, Carlsbad, USA) according to the manufacturer’s instructions. After a 48-h transfection, the cells were used for further experiments.

### Plasmid construction

pDonR223-FGF9, pDonR223-CCND2, and pDonR223-TGFBR1 plasmids carrying human FGF9, CCND2, and TGFBR1 genes were purchased from Changsha Axybio Bio-Tech Co., Ltd. (Changsha, China). The complete coding sequences of human FGF9, CCND2 and TGFBR1 were amplified from the pDonR223-FGF9, pDonR223-CCND2, and pDonR223-CCND2 plasmids, respectively. FGF9, CCND2, and TGFBR1 products and pEGFP-N1 plasmid were digested with XhoI and HindIII, and the fragments were purified and ligated with T4 DNA ligase. The ligated product was transformed into Top10 competent cells, and the positive clones were named pEGFP-N1- FGF9, pEGFP-N1- CCND2, and pEGFP-N1- TGFBR1.

### Quantitative real-time polymerase chain reaction

To evaluate the expressions of miR-4317, FGF9, CCND2, and TGFBR1, respectively, total RNA was used for qRT-PCR on a Step One Plus real-time system (AB Applied Biosystems, Carlsbad, CA, USA). U6 and GAPDH were used as internal controls. All primers used in this study are listed in Additional file 1: Table S1.

### Target prediction and luciferase reporter assays

Bioinformatics analysis was performed using miRDB (http://www.mirdb.org/), miRanda (http://www.microrna.org), and TargetScan (http://www.targetscan.org/). The 3’-UTRs of human FGF9, CCND2, and TGFBR1 were amplified from human genomic DNA and individually inserted into pmiR-RB-REPORT™ (Ribobio) via the XhoI and NotI sites. Similarly, the FGF9, CCND2, and TGFBR1 3’-UTR mutant fragments were inserted into the pmiR-RB-REPORT™ control vector at the same sites. For the reporter assays, NSCLC cells were co-transfected with wild-type reporter plasmid and miR-4317 mimics. Firefly and *Renilla* luciferase activities were measured in cell lysates using the dual-luciferase reporter assay system. Luciferase activity was measured 48 h post-transfection using the Dual-Glo Luciferase reporter system according to the manufacturer’s instructions (Promega, Madison, WI, USA). Firefly luciferase units were normalized against *Renilla* luciferase units to control for the transfection efficiency.

### In vitro cell proliferation assays

For the cell proliferation assays, cells were seeded into each well of a 96-well plate (5 × 10^3^ per well), and cell proliferation was determined by MTS (3-(4,5-dimethylthiazol-2-yl)-5-(3–carboxymethoxyphenyl)-2-(4-sulfophenyl)-2H-tetrazolium) according to the manufacturer’s instructions(Best Bio, China). MTS solution was added (20 μL/well) to each well and incubated at 37 °C for 2 h. The optical density of each sample was immediately measured using a microplate reader (Bio-Rad, Hercules, CA, USA) at 570 nm.

### Colony formation assay

Cells were transfected with miR-4317 mimic or miR mimic NC, miR-4317 inhibitor or miR inhibitor NC, as described above. Twenty-four hours later, the transfected cells were trypsinized, counted, and replated at a density of 1 × 10^3^ cells/10-cm dish. Ten days later, colonies resulting from the surviving cells were fixed with 3.7% methanol, stained with 0.1% crystal violet, and counted. Colonies containing at least 50 cells were scored. Each assay was performed in triplicate.

### Transwell migration/invasion assay

In vitro cell migration assays were performed according to the manufacturer’s instructions using transwell chambers (8 μM pore size; Costar, New York, USA). Cells were allowed to grow to subconfluency (~ 75–80%) and were serum-starved for 24 h. After detachment with trypsin, the cells were washed with PBS and resuspended in serum-free medium. Next, 100 μL of the cell suspension (5 × 10 ^4^ cells/mL) was added to the upper chamber. Complete medium was added to the bottom wells of the chambers. For screening, the cells that had not migrated after 24 h were removed from the upper face of the filters using cotton swabs, but the cells that had migrated were fixed with 5% glutaraldehyde solution to determine the number of migratory cells. The lower surfaces of the filters were stained with 0.25% Trypan Blue. Images of six different × 10 fields were captured from each membrane, and the number of migratory cells was counted. The mean of triplicate assays for each experimental condition was used. Similar inserts coated with Matrigel were used to evaluate the cell invasive potential in the invasion assay.

### Flow cytometry analysis

FACS analysis was performed 48 h post-transfection. The cells were harvested, washed with cold PBS, fixed in 70% ethanol at − 20 °C for 24 h, stained with 50 μg/mL propidium iodide (4ABio, China), and analyzed using a FACS Calibur flow cytometer (BD Biosciences, MA, USA). The results were analyzed using ModFit software (BD Biosciences). Three independent assays were conducted.

### Western blot analysis

For the western blot analyses, RIPA buffer containing protease inhibitors and phospha tase inhibitors (Roche, Basel, Switzerland) was used to prepare whole-cell lysates. Briefly, equal amounts of lysate were separated by SDS-polyacrylamide gel electrophoresis and then transferred to PVDF membranes (Millipore,Massachusetts, USA). After blocking with 5% bovine serum albumin, the membranes were probed with anti-FGF9 or CCND2 and anti-GAPDH (ab71395, ab226972, ab9485, Abcam, Cambridge, UK), followed by incubation with a horseradish peroxidase–conjugated secondary antibody [goat-anti-mouse IgG (1:2000) and goat-anti-rabbit IgG (1:3000)]. The proteins were visualized using Image Reader LAS-4000 (Fujifilm) and analyzed with Multi Gauge V3.2 software (GE Healthcare Life Sciences, USA).

### Generation of stable cell lines

Recombinant lentiviral vectors containing the miRNA-4317 knockdown and irrelevant sequence were purchased from Xibeihongcheng Biotechnology (Beijing, China). Added to the lentivirus expression vectors was a luciferase and puromycin reporter gene driven by the EF1α promoter to indicate the infection efficiency in a timely manner. To construct lentiviral vectors, the precursor sequences for the miRNA-4317 and irrelevant sequence (negative control) were inserted into pHBLV-U6-MCS-EF1α-Luc-T2A-puromycin lentiviral vectors. The recombinant lentiviruses were packaged by co-transfection of HEK-293 T cells with pSPAX2 and pMD2.G with LipoFiter™ reagent. The supernatant with lentivirus particles was harvested at 48 and 72 h after transfection and filtered through 0.45-μm cellulose acetate filters (Millipore). Recombinant lentiviruses were concentrated by ultracentrifugation. To establish stable cell lines, NSCLC cells were transduced with lentivirus at an MOI of approximately 5 in the presence of 5 μg/ml polybrene. The supernatant was removed after 24 h and replaced with fresh complete culture medium. The infection efficiency was confirmed by RT-PCR 96 h after infection, and the cells were selected with 2 μg/ml puromycin for 2 weeks.

### Tumorigenicity and metastasis assay in vivo

All animals received humane care in compliance with the “Guide for the Care and Use of Laboratory Animals” prepared by the Institute of Laboratory Animal Resources published by the National Institutes of Health and according to the Animal Experiment Guidelines of Samsung Biomedical Research Institute. The effect of miR-4317 on the tumorigenic and metastatic potential of NSCLC cells was analyzed in subcutaneous and systemic metastasis in vivo models via right subcutaneous tissue and tail vein injection, respectively. For the subcutaneous model, 4–6-week-old BALB/c nude mice received a subcutaneous injection of 1 × 10^6^ transfected cells in the right hip. For the experimental metastasis in vivo model, transfected cancer cells (1 × 10^6^ in 100 μL of HBSS) were injected directly into the tail vein. Six weeks later, the tumor colony in the subcutaneous tissue was observed by HE staining and histological examination. Bioluminescence images were collected to assess the growth and metastasis of the implanted tumor cells. To quantify the in vivo bioluminescence signal, the mice were anesthetized with isoflurane before in vivo imaging, and D-luciferin solution (in vivo imaging solutions, PerkinElmer, 150 mg/kg in PBS, Waltham, Massachusetts, USA) was injected intravenously for both subcutaneous and systemic xenografts. Bioluminescence images were acquired with the IVIS Spectrum imaging system (PerkinElmer,) 2–5 min after injection, and the captured images were quantified using the Living Image Software package (PerkinElmer/Caliper Life Sciences) by measuring the photon flux (photons/s/cm^2^/steradian) within a region of interest drawn around the bioluminescence signal.

### Agomir treatment

The agomir and the miRNA negative control were synthesized by the Bioribo Company and applied as per the manufacturer’s instructions. A 10-nmol miR-4317 agomir and the miRNA negative control in 0.1 ml saline buffer were locally injected into the NSCLC cell-forming tumor mass once every 5 d for 6 weeks. After the treatment, the NSCLC cell-forming tumors were applied for the immunohistochemistry assay. The tumor size was monitored by measuring the length (L) and width (W) with calipers every 5 d, and the volumes were calculated using the following formula: (L × W^2^)/2. Mice were euthanized by cervical dislocation on day 42, and the tumors were excised and snap-frozen for protein and RNA extraction.

### Evaluation of immunohistochemical staining

Sections were deparaffinized and boiled in 10 mM citrate buffer (pH 6.0) for antigen retrieval. Endogenous peroxidase was blocked with 3% H_2_O_2_. The slides were incubated with primary antibodies, including Ki-67 (1:50, ab156956, Abcam), FGF9 (1:50), and CCND2 (1:50), respectively, followed by HRP-labeled secondary antibody. They were then visualized with diaminobenzidine. The expression levels of FGF9 and CCND2 in cytoplasm were determined by the product score of the average percentage and intensity of positive cells under 5 random high-power fields. Scores for percentage: < 5% (0), 5–25% (1), 25–50% (2), 50–75% (3), and > 75% (4); scores for intensity: no staining (0), light brown (1), brown (2), and dark brown (3). For FGF9 and CCND2, scores of 0 and ≥ 1 were defined as negative and positive, respectively.

### Statistical analysis

All measurement data are expressed as means ± standard deviation. Error bars represent the standard errors of the means. Student’s *t* test, χ^2^ test and repeated measures ANOVA were used to perform statistical comparisons. Log-rank test was used to analyze the effect of clinical variables and miRNAs on OS of patients. Multivariate Cox regression models were used to assess factors associated with OS in NSCLC. Receiver operating characteristic curves and the area under the curve were used to assess the feasibility of using serum miRNA as a diagnostic tool for detecting NSCLC. *p* < 0.05 was considered statistically significant. Statistical analyses were performed using SPSS 16.0 software (SPSS Inc., USA).

## Results

### Identification of miR-4317 in NSCLC

**Fig. 1:**
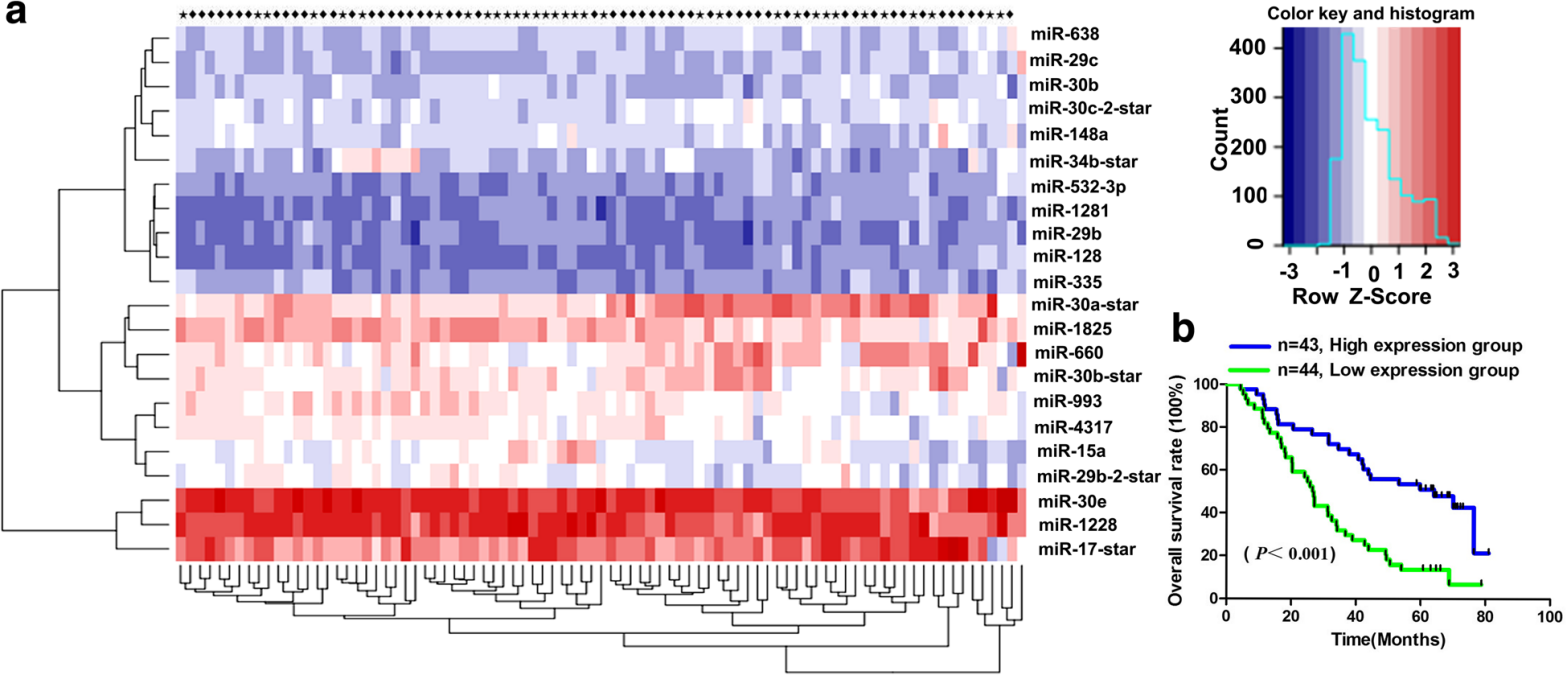
Clustering of miRNA expression profiling. **a** Clustering of miRNA expression profiling of 32 lung adenocarcinoma cases that developed BM and 55 BM-free cases. **b** Kaplan-Meier overall survival curves by high and low miR-4317 expression in 87 NSCLC patient cases with lung adenocarcinoma

**Fig. 2:**
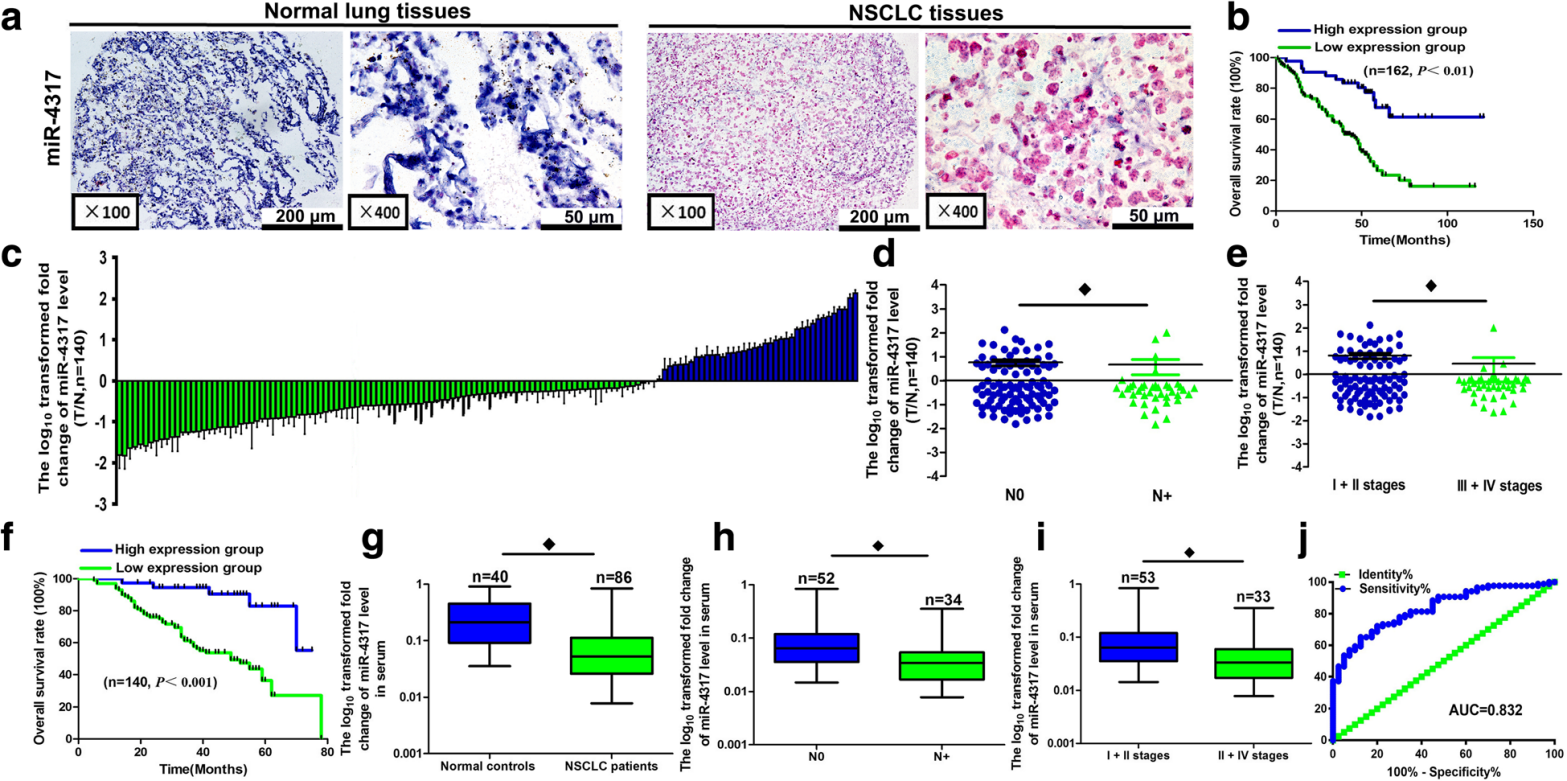
Relative miR-4317 expression levels in NSCLC tissues and serum and its clinical significance. **a** Expression levels of miR-4317 in 162 paired NSCLC and corresponding noncancerous tissues measured by in situ hybridization. **b** Kaplan-Meier overall survival curves by high and low miR-4317 expression in 162 NSCLC patients. **c** Quantitation of miR-4317 was performed using qRT-PCR in 140 paired NSCLC (T) and corresponding normal tissues (N). The fold changes were calculated by relative quantification (2^−∆Ct^, U6 as the internal control). **d**, **e** miR-4317 expression was detected in lymph node metastasis (**d**) and different clinical stages (**e**) of NSCLC. **f** Kaplan-Meier curves depicting overall survival according to the expression of miR-14,317 as validation. **g** The expression level of serum miR-4317 in 86 NSCLC patients and 40 healthy controls was measured by qRT-PCR and normalized to U6. **h**, **i** miR-4317 expression was detected in lymph node metastasis (**h**) and different clinical stages (**i**). **j** Receiver-operating characteristic curve analysis of the miR-4317 assay ratio for detecting NSCLC patients. ♦*p* < 0.05

To identify miRNAs associated with OS in lung adenocarcinoma, we analyzed miRNA expression profiles in formalin-fixed paraffin-embedded specimens of 87 lung adenocarcinoma patients according to the expression level of each miRNA among 309 detected human microRNAs. We found that 22 miRNAs were significantly associated with the OS of NSCLC patients, among which miR-4317 could distinguish the survival status of NSCLC patients (Fig. 1a and b). Since the role of miR-4317 in tumor progression had never been reported, in this study, we characterized miR-4317 expression in NSCLC. To further explore the expression of miR-4317 in tumor tissue samples, 162 pairs of NSCLC and matched normal lung tissues were analyzed by in situ hybridization (ISH). The results showed that miR-4317 was significantly downregulated in tumor tissues, including adenocarcinoma tissues and squamous cell carcinoma tissues (Fig. 2a). Kaplan-Meier survival analysis showed that miR-4317 expression was significantly associated with the OS of NSCLC patients. Patients with high miR-4317 expression had better OS compared to those with low expression (*n* = 162; *p* < 0.01; Fig. 2b). Furthermore, a lower level of miR-4317 was detected in advanced clinical stages and during lymph node metastasis compared with early clinical stages and in the presence of non-metastatic lymph nodes (*p* < 0.003; Table 1). There was no difference in age, gender, tumor size, pathologic classification, differentiation, or local invasion (*p* > 0.05; Table 1). Multivariate Cox regression analysis showed that a low expression level of miR-4317 was an independent prognostic factor for poor OS of NSCLC (RR = 0.29; *p* = 0.001; Table 3).

**Table 3:** Multivariate Cox regression analysis of factors associated with OS in NSCLC

Factor	Training group (*n* = 162)			Test group (*n* = 140)		
	95% CI	*RR*	*p*	95% CI	*RR*	*p*
Sex (male vs. female)	0.43–1.41	0.78	0.40	0.75–3.41	1.60	0.22
Age (≤ 60 y vs. > 60 y)	0.73–2.06	1.23	0.44	0.83–2.80	1.52	0.18
Tumor size (≤ 5 cm vs. > 5 cm)	0.75–2.39	1.34	0.33	0.40–1.61	0.81	0.54
Tumor stages (T1 + T2 vs. T3 + T4)	0.40–1.53	0.79	0.48	0.71–3.54	1.58	0.26
Histological type (adenocarcinoma vs. squamous cell carcinoma)	0.31–1.07	0.57	0.08	0.40–1.80	0.85	0.68
Histologic grade (well/moderate vs. poor/NS)	0.61–1.90	1.08	0.80	0.62–2.11	1.14	0.68
Lymph node metastasis (negative vs. positive)	0.77–3.32	1.63	0.18	0.96–4.21	2.01	0.06
Clinical stages (I + II vs. III + IV)	0.73–4.50	3.73	0.23	0.63–3.46	1.48	0.37
miR-4317 expression level (low vs. high)	0.14–0.58	0.29	0.001	0.09–0.63	0.24	0.004

To validate the downregulation of miR-4317 in NSCLC, quantitative reverse transcription polymerase chain reaction (qRT-PCR) was performed in 140 pairs of NSCLC lung tissues and matched normal lung tissues. The results showed that the miR-4317 levels were significantly lower in NSCLC lung tissues than in normal lung tissues (Fig. 2c), especially in advanced clinical stages and during lymph node metastasis of NSCLC tissues with reduced expression levels (Fig. 2d and e; Table 1). Kaplan-Meier survival analysis also revealed that downregulated miR-4317 was associated with a poor prognosis in patients with NSCLC (*n* = 140; *p* < 0.001; Fig. 2f). Multivariate Cox regression analysis showed that a low expression level of miR-4317 was an independent prognostic factor for poor OS of NSCLC (RR = 0.24; *p* = 0.004; Table 3).

Finally, to confirm whether miR-4317 could be a potential noninvasive biomarker for NSCLC, we investigated the serum expression of miR-4317 in 86 patients with NSCLC and 40 healthy controls by qRT-PCR. The results showed that serum expression levels of miR-4317 were significantly lower in patients with NSCLC than in healthy controls (Fig. 2g). A low serum expression level of miR-4317 was significantly correlated with lymph node metastasis and advanced clinical stages (*p* < 0.004; Fig. 2h and i; Table 2). No significant correlations were found between serum expression levels of miR-4317 and age, gender, tumor size, pathologic classification, differentiation, or local invasion (*p* > 0.05; Table 2). The area under the curve for plasma miR-4317 was 0.832 (Fig. 2j).

### miR-4317 inhibited the malignant phenotype of NSCLC in vitro

**Fig. 3:**
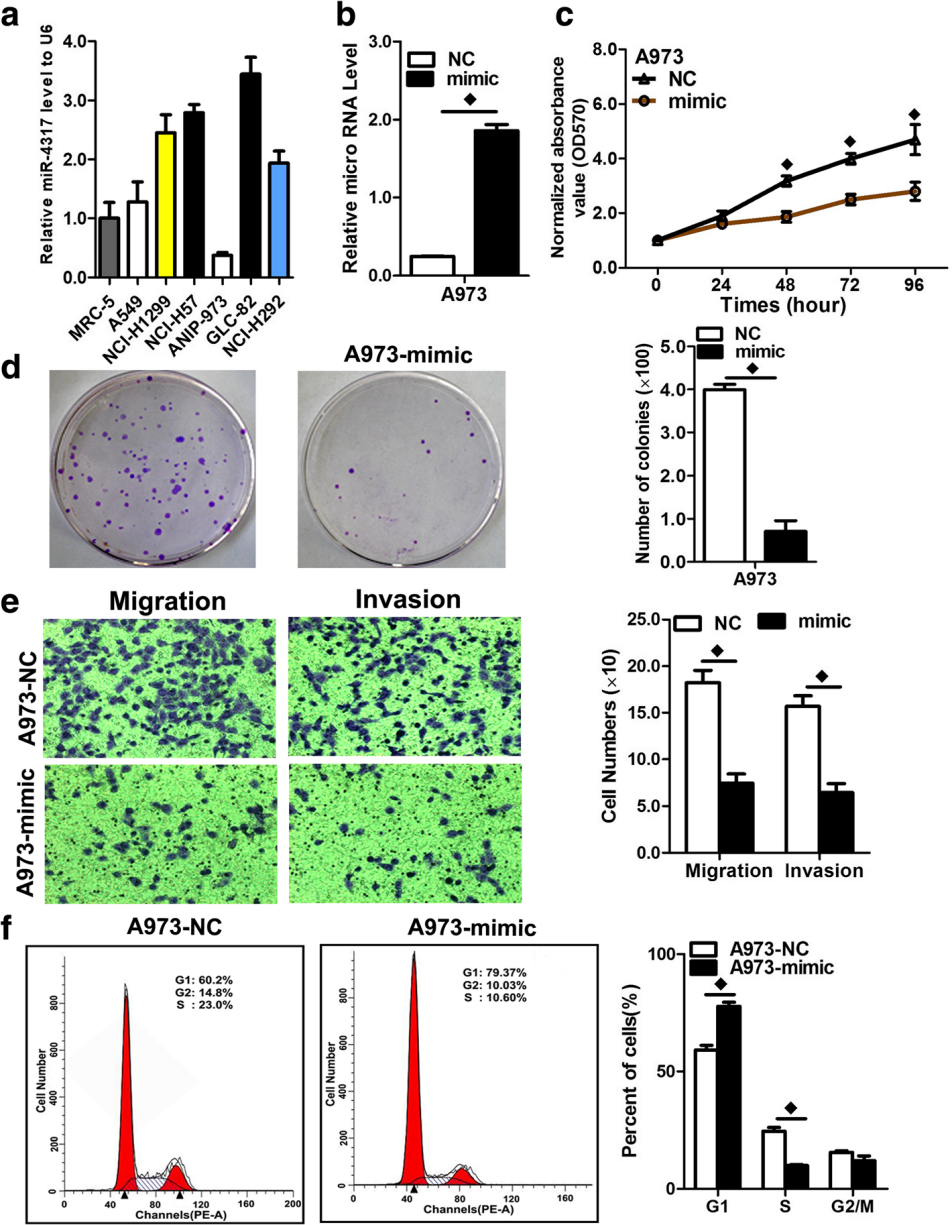
miR-4317 overexpression inhibited cell proliferation, colony formation, and migration. **a** RNA level of miR-4317 in 6 NSCLC cell lines. **b** Quantitation of the miR-4317 level after transfection of miR-4317 mimic in A973 cell lines. **c** The cell growth curve was measured by MTS after transfection of the miR-4317 mimic in A973 cell lines, and the OD_570_ was normalized to the start point (0 h). **d** Representative images and quantitation of colony formation were performed after transfection of miR-4317 mimic in A973 cell lines. **e** Representative images and quantitation of the transwell assay were performed after transfection of the miR-4317 mimic in A973 cell lines. **f** miR-4317 induced cell cycle arrest at the G1/S phase. Data are presented as the mean values ± SD from triplicate experiments. ♦*p* < 0.05

To assess the role of miR-4317 in NSCLC, we initially tested the miR-4317 levels in a normal human embryo lung fibroblast cell line (MRC-5) and 6 NSCLC cell lines (Fig. 3a). miR-4317 was decreased the most in A973 and A549 cells, but was increased in GLC82 and H157 cells. Thus, we chose these four cell lines to perform the following experiments.

The efficiency of mimic transfection was verified by a significant increase in miR-4317 expression in A973 and A549 cells, as determined by qRT-PCR (Fig. 3b; Additional file 2: Figure S1a). We observed that the exogenous high expression level of miR-4317 remarkably inhibited proliferation, colony formation, migration, and invasion of A973 and A549 cells (Fig. 3c, d, and e; Additional file 2: Figure S1b, c, and d). To explore the possible mechanism underlying the inhibitory effect on cell growth by overexpression of miR-4317, cell cycle analysis was performed. Upon upregulation of miR-4317, the percentages of A973 and A549 cells in G0/G1 phase clearly increased compared with the controls (Fig. 3f; Additional file 2: Figure S1e), indicating that overexpression of miR-4317 resulted in G1 phase arrest in NSCLC cells.

**Fig. 4:**
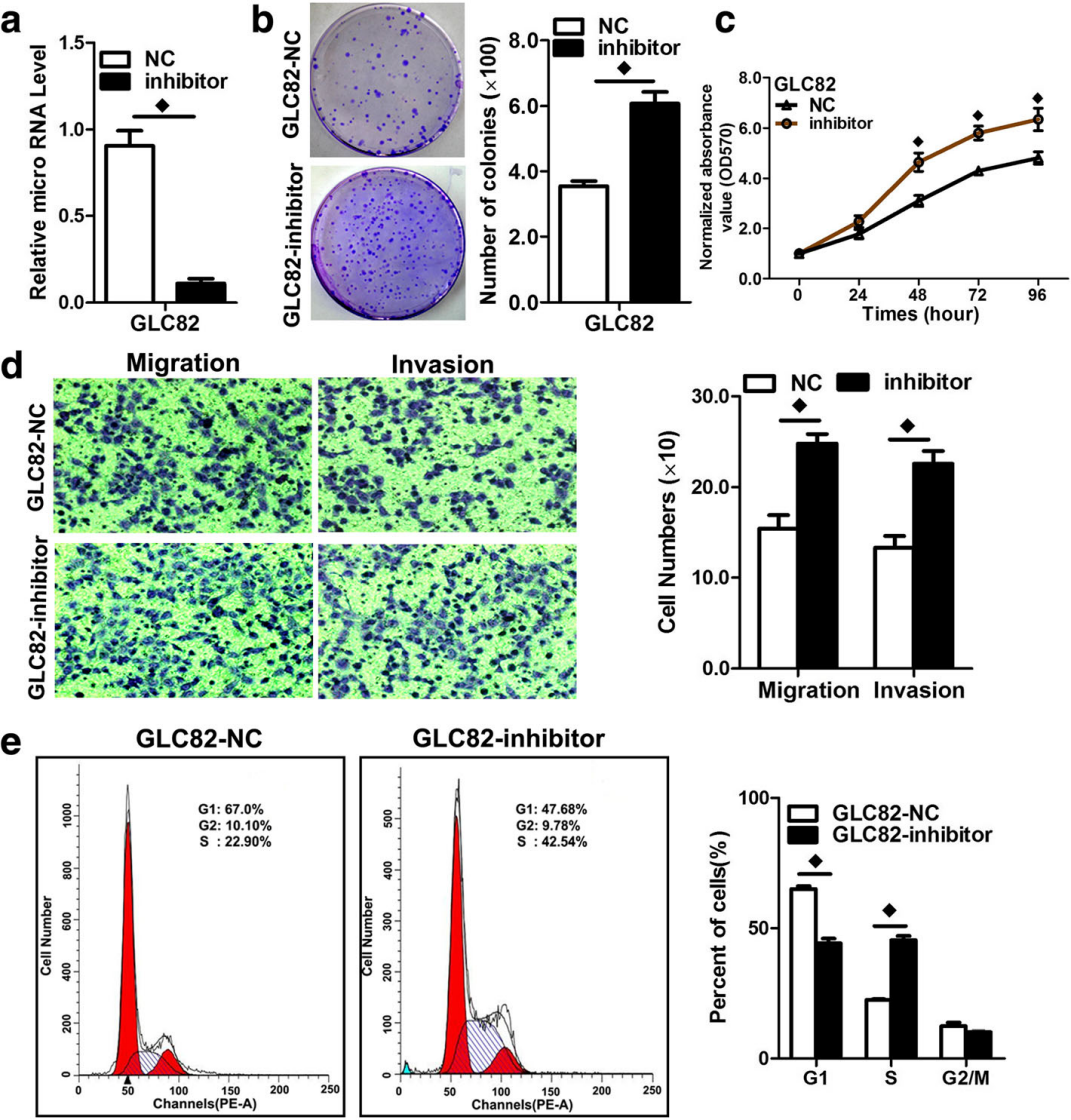
Repression of miR-4317 expression significantly promoted cell growth, colony formation, and migration in GLC82 cells. **a** Quantitation of the miR-4317 level after transfection of the miR-4317 inhibitor in GLC82 cell lines. **b** Representative images and quantitation of colony formation were performed after transfection of the miR-4317 inhibitor in GLC82 cell lines. **c** The cell growth curve was measured by MTS after transfection of the miR-4317 inhibitor in GLC82 cell lines, and the OD_570_ was normalized to the start point (0 h). **d** Representative images and quantitation of the transwell assay were performed after transfection of the miR-4317 inhibitor in the GLC82 cell lines. **e** miR-4317 induced cell cycle arrest at G1/S phase. Data are presented as the mean values ± SD from triplicate experiments. ♦*p* < 0.05

We also transfected NSCLC cells with inhibitors of miR-4317 to confirm the opposite results of mimic transfection (Fig. 4a; Additional file 3: Figure S2a). As expected, downregulation of miR-4317 using inhibitors enhanced the malignant phenotypes of GLC82 and H157 cells in vitro, including colony formation (Fig. 4b; Additional file 3: Figure S2b), cell growth (Fig. 4c; Additional file 3: Figure S2c), and cell migration and invasion (Fig. 4d; Additional file 3: Figure S2d). We further found that down-expression of miR-4317 decreased the proportion of cells in G1 phase and increased the proportion in S phase (Fig. 4e; Additional file 3: Figure S2e), indicating that miR-4317 downregulation could accelerate the conversion from G1 phase to S phase in both GLC82 and H157 cells.

### miR-4317 targeted fibroblast growth factor 9 (FGF9) and cyclin D2 (CCND2) to suppress NSCLC proliferation and metastasis

**Fig. 5:**
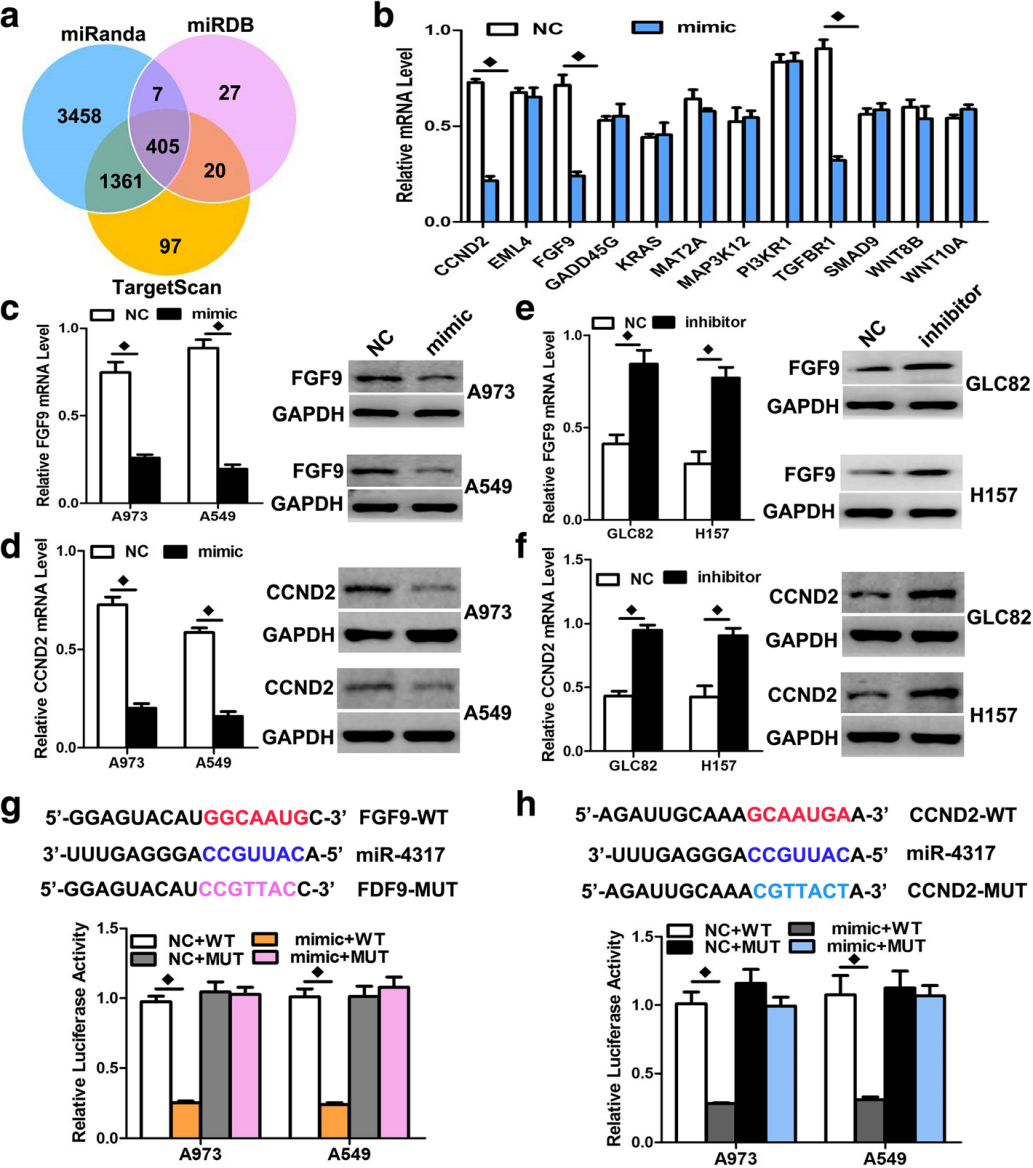
FGF9 and CCND2 were two direct target genes of miR-4317. **a**, **b** TGFBR1 and CCNE2 were identified as potential regulatory targets of miR-4317 by considering the downregulation of genes using prediction tools and qRT-PCR. **c**, **d** The expression levels of FGF9 and CCND2 mRNA and protein were measured by qRT-PCR and western blot analysis using GAPDH as the loading control after transfection of miR-4317 mimic in the A973 and A549 cell lines, respectively. **e**, **f** The expression levels of FGF9 and CCND2 mRNA and protein were measured by qRT-PCR and western blot analysis using GAPDH as the loading control after transfection of miR-4317 inhibitors in the GLC82 and H157 cell lines, respectively. **g**, **h** Dual-luciferase reporter assay. The relative luciferase activity was normalized to the *Renilla* luciferase activity assay after co-transfection of cells with miR-4317 mimic and pmiR-RB-REPORT™ constructs containing WT or MUT FGF9 and the CCND2 3’-UTR region in A973 and A549 cell lines. Data are presented as the mean values ± SD from triplicate experiments. ♦*p* < 0.05

To explore the mechanism by which miR-4317 regulates NSCLC cell progression, we searched for potential regulatory targets of miR-4317 using several bioinformatics methods, including TargetScan, miRDB, and miRanda (Fig. 5a). In total, 405 genes were simultaneously predicted by the three databases, and FGF9, CCND2, and TGFBR1 were identified as candidate genes with relevance to NSCLC based on their associated Gene Ontology terms.

To verify whether FGF9 and CCND2 are direct targets of miR-4317, miR-4317 mimic was transfected into cells and was found to markedly downregulate the mRNA and protein levels of FGF9 and CCND2, respectively (Fig. 5b, c, d, e, and f). We next applied the dual-luciferase reporter assay to reveal the manner by which miR-4317 regulates FGF9 and CCND2. The fragments containing the miR-4317 binding sequence or mutated sequence in the 3’UTRs of FGF9 and CCND2 were cloned into the pmiR-RB-REPORT™ vector luciferase reporter. These reporter constructs were co-transfected with miR-4317 mimic or miRNA negative control into A973 and A549 cells, and the luciferase activities were subsequently measured. The miR-4317 mimic significantly suppressed the luciferase activity of pmiR-RB-REPORT™-FGF9 and CCND2–3’UTR (Fig. 5g and h), while the miRNA negative control had no inhibitory effect on pmiR-RB-REPORT™-FGF9 or CCND2–3’UTR. The miR-4317 inhibition of pmiR-RB-REPORT™-FGF9 and CCND2–3’UTR was sequence-specific because the luciferase activities of pmiR-RB-REPORT™-FGF9 or CCND2-mut did not reveal any reductions in the presence of miR-4317. Although miR-4317 mimic can downregulate the mRNA level of TGFBR1, the miR-4317 mimic did not significantly suppress the luciferase activity of pmiR-RB-REPORT™-TGFBR1–3’UTR (Additional file 4: Figure S3). Together, these results suggested that miR-4317 directly targets the 3’-UTRs of FGF9 and CCND2.

**Fig. 6:**
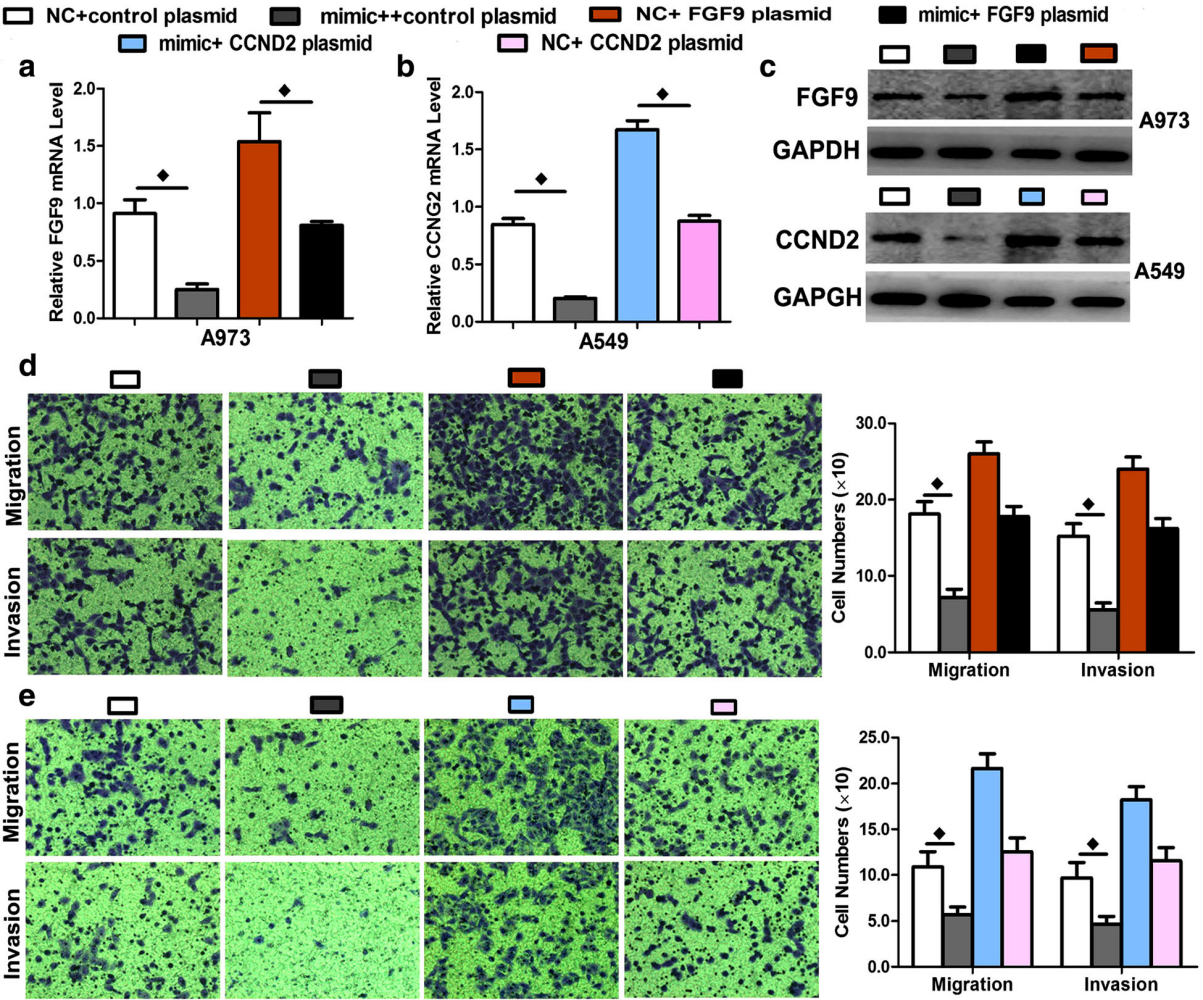
Rescue assay to confirm whether FGF9 and CCND2 were the functional targets of miR-4317. **a-c** The mRNA and protein levels of FGF9 and CCND2 in A973 and A549 cell lines co-transfected with miR-4317 mimic and pEGFP-C1 plasmid containing FGF9 and CCND2 CDS sequence. **d-e** Transwell assay of cells co-transfected with miR-4317 mimic and FGF9 and CCND2 plasmids. Data are presented as the mean values ± SD from triplicate experiments. ♦*p* < 0.05

A rescue experiment was performed to confirm that FGF9 and CCND2 were functional targets of miR-4317 in A973 and A549 cells. FGF9 and CCND2 mRNA and protein (endogenous) in the two cell lines were abolished by mimic transfection and recovered by transfection of both pEGFP-N1-FGF9 and CCND2 expression constructs, respectively (Fig. 6a, b, and c). The results showed that migration and invasion created by mimic transfection were reversed by transfection of both expression constructs (Fig. 6d and e).

### miR-4317 suppressed tumor growth and metastasis in vivo

**Fig. 7:**
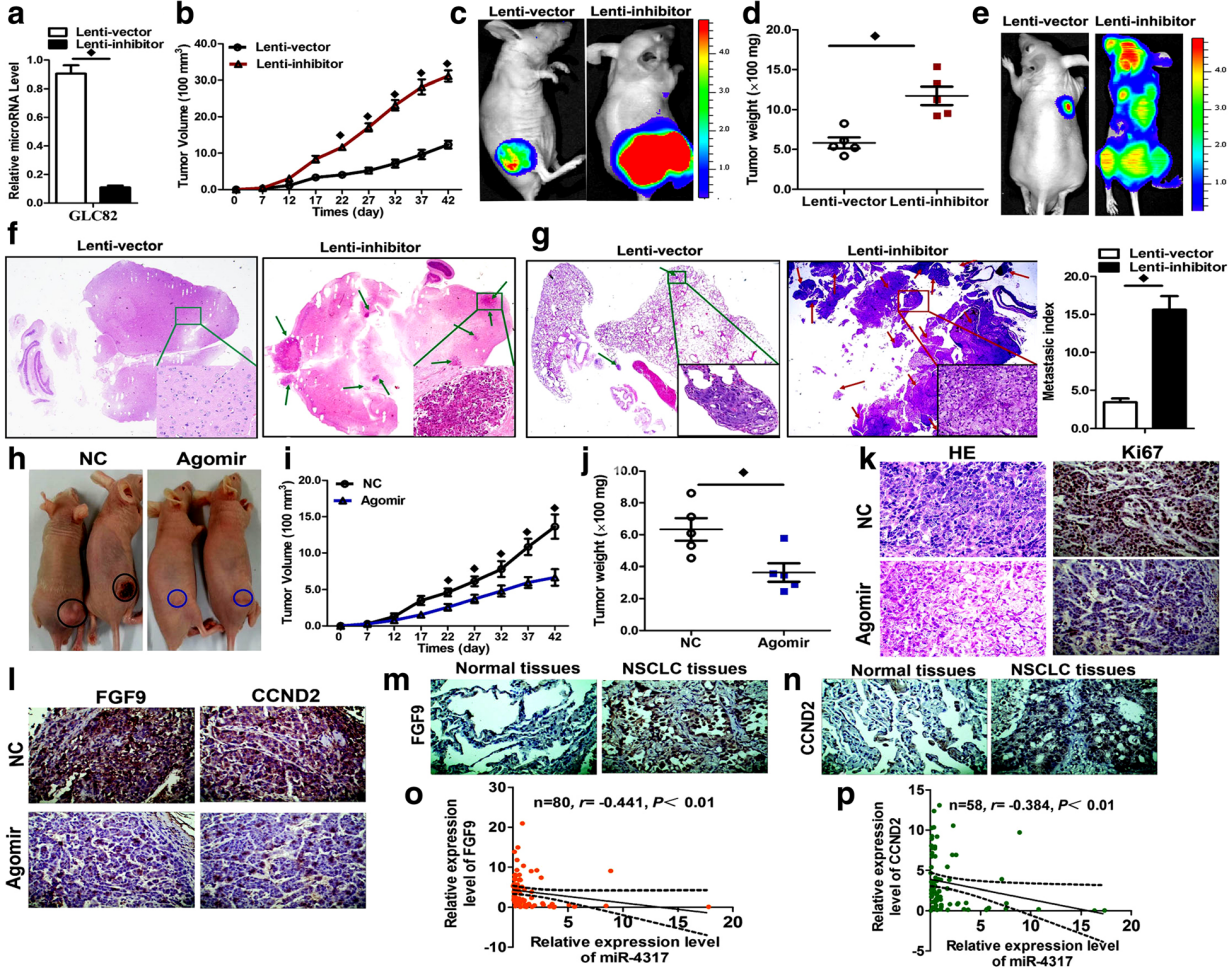
miR-4317 suppressed tumor growth and metastasis in vivo. **a** Levels of miR-4317 in stable miR-4317 knockdown GLC82 cells (Lenti-inhibitor) and control GLC82 cells (Lenti-vector). **b-d** Stable miR-4317 knockdown GLC82 cells were subcutaneously injected into nude mice to form solid tumors, and representative images of tumor volumes and weights were analyzed by in vivo luciferase imaging on the last day of analysis (*n* = 5 for each group). **e**-**g** The number of metastatic nodules was observed and quantified in the brains and lungs of mice treated with stable miR-4317 knockdown GLC82 cells or control vector cells by the vein injection method. **h-j** GLC82 cells were subcutaneously injected into nude mice to form solid tumors and synchronously treated with miR-4317 agomir or miR agomir NC (*n* = 5 for each group); a 10-nmol miR-4317 agomir as well as the miRNA negative control in 0.1 ml saline buffer were locally injected into nude mice to form a tumor mass once every 5 days for 6 weeks; tumor weight and volume were measured. **k**, **l** Immunohistochemical staining of Ki67, FGF9, and CCND2 in tumor tissues dissected from nude mice treated with miR-4317 agomir miR or agomir NC. **m**, **n** FGF9 and CCND2 protein expression measured by immunohistochemical staining in 80 NSCLC samples and pair-matched lung tissues. **o**, **p** Spearman correlation analysis of the negative correlation between the expression of miR-4317 and FGF9/CCND2. ♦*p* < 0.05

We evaluated the effects of miR-4317 on the growth and metastasis of NSCLC in nude mice. First, GLC82 cells were transfected with either a lentiviral expression vector to knock-down miR-4317 or a negative control lentiviral vector. Efficient downregulation of miR-4317 in GLC82 cells following lentiviral infection was verified by qRT-PCR (Fig. 7a). Next, we subcutaneously injected these GLC82 cells into mice to generate transplanted tumors of BALB/c nude mice. Beginning on day 7 after implantation, the tumor lengths and widths were measured every 5 days to collect 8 measurements. The tumor growth curve revealed a significant acceleration in the miR-4317-downregulated group compared with the control group (Fig. 7b). Subsequently, the tumors were dissected, and the exact sizes and weights were evaluated. Compared with the control group, the mean volume and mass of the tumors in the miR-4317 downregulated group were significantly larger and heavier (Fig. 7c and d).

Mice received 10^6^ luciferase-labeled cells injected intravenously into the tail vein and were observed for 6 weeks. Luciferase activity was used to evaluate tumor burden in the lung and brain. As expected, miR-4270 knockdown significantly increased the brain metastasis ratio of NSCLC cells through tail vein injection (Fig. 7e and f). Lung metastasis burden was significantly increased in mice that received an injection of cells with miR-4270 knockdown compared with those in the control group (Fig. 7e and g). Together, these results in mouse models suggest that miR-4270 plays important roles in the in vivo growth and metastasis of NSCLC, especially in the process of lung and brain metastasis.

To examine whether miR-4317 could inhibit the growth of NSCLC in nude mice, we established a BALB/c nude mouse tumorigenic and metastatic model using A973 cells. After 7 days, miR-4317 agomir or miR agomir NC was directly injected into the implanted tumor and tail vein eight times every 5 days. The tumor volume was measured every 5 days until day 42. The tumor volume and weight of the mice treated with miR-4317 agomir were significantly increased relative to those that received the miR agomir NC (Fig. 7h, i, and j), which indicated that miR-4317 significantly inhibited the tumorigenicity and metastasis of NSCLC cells in the nude mouse model.

Additionally, the proliferative activities of the tumor cells were assessed via immunohistochemical staining for Ki-67 in FFPE tissues of xenograft tumors. The Ki-67 staining intensities were decreased in the tumors from the miR-4317 agomir group (Fig. 7k). Moreover, there was an obvious decrease in FGF9 and CCND2 expression in IHC slices in the miR-4317 agomir group compared with the control group (Fig. 7l). In an analysis of 80 paired tumor and adjacent nontumor tissue samples, FGF9 and CCND2 expressions were significantly elevated in tumor tissues compared to adjacent nontumor tissues (Fig. 7m and n). We also conducted a Spearman correlation coefficient analysis to evaluate the association of the miR-4317 expression level and FGF9 or CCND2 expression in 80 NSCLC tissue samples. The miR-4317 expression levels were inversely correlated with the levels of FGF9 and CCND2 upregulation (Fig. 7o and p).

## Discussion

In this study, we evaluated the role of miR-4317 in lung carcinoma, as well as the underlying molecular mechanisms. First, we found that as a novel antitumor miRNA in NSCLC, miR-4317 was significantly downregulated in lung cancer tissues and exhibited expression levels that were inversely correlated with the clinical tumor stage and lymph node metastasis by ISH and qRT-PCR. Recently, miRNAs linked to the clinical tumor stage and lymph node metastasis of NSCLC have been identified, such as miR-10, miR-195, miR-338-3p, and miR-708-5p [18, 28–30]. Endogenous circulating miRNAs have attracted significant attention regarding the diagnosis, prognosis, and metastasis of cancer. Importantly, in the present study, the serum miR-4317 level was demonstrated to be useful in delineating lung cancer stages due to the decreasing expression of miR-4317 in higher-stage cancers. Lymph nodes that contained metastatic tumors also showed downregulation of miR-4317 expression. To the best of our knowledge, this is the first report on the diagnostic and prognostic value of serum miR-4317. Diagnostic tests that involve noninvasive procedures, such as a simple blood draws, are highly desirable and patient friendly. A decreased level of serum miR-4317 could be used to detect lung cancer early and accurately to determine lung cancer aggressiveness. This serum-based approach is more advantageous than biopsy, an invasive procedure that serves as the main tool for lung cancer risk assessment. miRNAs have also been extensively investigated as prognostic factors [31]. Our results showed that the high expression of miR-4317 might be closely associated with improved OS in NSCLC. Intriguingly, Zhou et al. demonstrated that a high expression level of miR-574-5p in serum was an independent poor prognostic risk factor in patients with SCLC [32]. Therefore, our findings demonstrate that miR-4317 may play a tumor-inhibiting role in NSCLC and prompted us to investigate its exact functions.

To better understand the underlying role of miR-4317 in lung cancer, we explored the biological function of miR-4317 in vitro and in vivo. In the present study, we showed that overexpression of miR-4317 inhibited the proliferation, colony formation, migration, and invasion capacity of NSCLC cells. In contrast, downregulation of miR-4317 had the opposite effects. Moreover, dysregulation of miR-4317 induced tumorigenesis and lung and brain metastases in nude mice. These results indicate that miR-4317 plays a crucial role in the growth and metastasis of NSCLC. Metastasis-related death accounts for approximately 90% of cancer mortality [33]. Accumulating evidence shows that miRNAs participate in tumor growth and metastatic processes, and a growing number of miRNAs have been found to be involved in lung cancer metastasis [34, 35]. Joshi et al. demonstrated that miR-148a might act as a tumor suppressor, and it inhibits migration and invasion of the A549 NSCLC cell line [36]. Upregulation of miR-150 results in a significant increase in tumor cell metastasis in vitro and lung metastases in a nude mouse xenograft model [37]. Similarly, the inhibitory roles of various miRNAs in tumor growth and metastasis have been demonstrated in previous NSCLC studies [30, 38, 39]. Furthermore, upregulation of miR-26b leads to inhibition of gastric cancer cell migration and invasion in vitro and lung metastasis formation in vivo [40, 41].

The mechanism by which miRNAs alter gene expression remains controversial, but most studies suggest that miRNAs are primarily processed by the RNA-mediated interference machinery to trigger partial or complete target gene mRNA degradation [42]. Our bioinformatics analysis revealed that miR-4317 could bind to the 3’UTRs of FGF9 and CCND2, and we observed that the expressions of FGF9 and CCND2 were repressed by miR-4317. FGF9, which is also known as glial activating factor, is one of 23 members of the highly conserved FGF family. As a secreted, glycosylated 26-kDa protein, it has mitogenic effects on a variety of cell types [43]. FGF9 is also required during lung development for mesenchymal growth and epithelial branching, and inactivation of FGF9 in mice results in perinatal death due to respiratory insufficiency [44–46]. FGF9 has been shown to be implicated in cancers, such as ovarian endometrioid adenocarcinoma [43], hepatocellular carcinoma [47], and prostate carcinoma [48]. Hendrix et al. showed that FGF9 possesses oncogenic activity [43]. Abdel-Rahman et al. confirmed that FGF9 activates a major intracellular effector of ERK MAP kinase [49]. Deng et al. identified a novel mechanism of miR-26a in the suppression of gastric cancer growth and metastasis by direct targeting of FGF9 protein expression [50]. miR-182 downregulation induces SMC differentiation, proliferation, and migration, and these processes are prevented by FGF9 knockdown; moreover, inhibition of FGF9 itself is able to suppress the dedifferentiation, proliferation, and migration of rat SMCs [51]. Importantly, Suzuki et al. examined FGF9 concentrations in lung cancer patient serum by ELISA assay and found that the mean concentration was less than the detectable range in 15 patients with lung cancer and 8 with other lung diseases [52]. Although FGF9 serum concentrations may not directly reflect the FGF9 concentration in specific patients with lung cancer, combining the above in vitro findings, we speculate that, if finding elevated levels of FGF9 in the blood of patients or mice with low miR-4317 would give further support to the conclusions of this article.

In this study, another new direct and functional target of miR-4317, CCND2, was also identified. CCND2 is a well-known cyclin that functions in the cell cycle, specifically in G1/S transition. We further conducted fluorescence activated cell sorting (FACS) analysis to confirm the role of miR-4317 as a negative regulator of the cell cycle and found that increased expression of this miRNA resulted in significant G0/G1 arrest and S phase reduction. Recent reporter assays have shown that CCND2 is targeted by let-7a, and that this interaction inhibits proliferation in human prostate cancer cells both in vitro and in vivo [53]. Moreover, overexpression of CCND2 results in excessive proliferation in many cell types and enhances OSCC cell invasive ability in vitro and in vivo [54]. In this study, we confirmed that FGF9 and CCND2 were direct targets of miR-4317 in NSCLC cells. To determine whether miR-4317 could suppress NSCLC growth and metastasis by repressing FGF9 and CCND2 expression, we found that FGF9 and CCND2 overexpression could rescue the invasion and growth defects of miR-4317. These results suggested that miR-4317 inhibited NSCLC growth and metastasis partly by targeting FGF9 and CCND2. However, some limitations remain in our study, in that FGF9 and CCND2 might be not the only two proteins influenced by miR-4317. Moreover, other miRNAs may regulate the expression of FGF9 and CCND2 in addition to miR-4317 in NSCLC. Therefore, our future work will be dedicated to examining these issues, specifically by knocking-down FGF9 or CCND2 to abolish the effects of miR-4317 transfection and investigating the relationship between other signaling pathways and FGF9 or CCND2 protein.

Additionally, the proliferative activities of the tumor cells were assessed via immunohistochemical staining for Ki-67. The Ki-67 staining intensities were decreased in tumors from the miR-4317-downregulated group. The results further revealed reduced FGF9 and CCND2 staining intensities in xenograft tumors in the miR-4317 agomir group compared with the NC group, indicating a decrease in tumor cell proliferation in response to the downregulation of miR-4317. Finally, we found that high expression levels of FGF9 and CCND2 in NSCLC tissues were inversely associated with miR-4317. However, there have been no reports concerning the relationship between FGF9 and CCND2. Intriguingly, a recent study suggested that miR-4317 could repress the proliferation of gastric cancer cells by targeting and suppressing ZNF322 [55].

## Conclusions

In summary, we observed the downregulation of miR-4317 in NSCLC tissues and demonstrated that miR-417 might act as an independent predictor for OS of NSCLC. Moreover, serum miR-4317 may be used as a novel and stable marker for NSCLC. We further found that miR-4317 has the potential to suppress NSCLC growth and metastasis and to induce cell cycle arrest by regulating FGF9 and CCND2. Our findings suggest that miR-4317 functions as a tumor suppressor in NSCLC and holds promise as a prognostic biomarker and potential therapeutic target for NSCLC.

## Additional files

Additional file 1: **Table S1.** Specific primers. Specific primers used in this study. (DOC 29 kb)

Additional file 2: **Figure S1.** miR-4317 overexpression inhibited cell proliferation, colony formation, and migration. a Quantitation of the miR-4317 level after transfection of miR-4317 mimic in A549 cell lines. b The cell growth curve was measured by MTS after transfection of miR-4317 mimic in A549 cell lines, and the OD_570_ was normalized to the start point (0 h). c Representative images and quantitation of colony formation after transfection of miR-4317 mimic in A549 cell lines. d Representative images and quantitation of the transwell assay after transfection of miR-4317 mimic in A549 cell lines. e miR-4317 induced cell cycle arrest at G1/S phase. Data are presented as the mean values ± SD from triplicate experiments. ♦*p* < 0.05. (JPG 86 kb)

Additional file 3: **Figure S2.** Repression of miR-4317 expression significantly promoted cell growth, colony formation, and migration in H157 cells. a Quantitation of the miR-4317 level after transfection of miR-4317 inhibitor in H157 cell lines. b The cell growth curve was measured by MTS after transfection of miR-4317 inhibitor in H157 cell lines, and the OD_570_ was normalized to the start point (0 h). c Representative images and quantitation of colony formation after transfection of miR-4317 inhibitor in H157 cell lines. d Representative images and quantitation of the transwell assay after transfection of miR-4317 inhibitor in H157 cell lines. e miR-4317 induced cell cycle arrest at G1/S phase. Data are presented as the mean values ± SD from triplicate experiments. ♦*p* < 0.05. (JPG 590 kb)

Additional file 4: **Figure S3.** Dual-luciferase reporter assay. The relative luciferase activity was normalized to the *Renilla* luciferase activity assay after co-transfection of cells with miR-4317 mimic and pmiR-RB-REPORT™ constructs containing WT or MUT TGFBR1 3’-UTR in A973 and A549 cell lines. (JPG 639 kb)

## Abbreviations

APC: Adenomatous polyposis coli
AUC: ROC curve
BSA: Bovine serum albumin
EMT: Epithelial-mesenchymal transition
FACS: Fluorescence activated cell sorting
HEK: Human embryonic kidney
ISH: In situ hybridization
miRNAs: MicroRNAs
NC: Negative controls
NSCLC: Non-small cell lung cancer
OS: Overall survival
ROC: Receiver operating characteristic
ROI: Region of interest
RT: Reverse transcription
SDS-PAGE: SDS-polyacrylamide gel electrophoresis
