# Supplementary material for: miR-4317 suppresses non-small cell lung cancer (NSCLC) by targeting fibroblast growth factor 9 (FGF9) and cyclin D2 (CCND2)
Source: J Exp Clin Cancer Res. 2018 Sep 18;37:230. doi: 10.1186/s13046-018-0882-4 (PMC6145328; doi:10.1186/s13046-018-0882-4)
Supplement: Supplementary file 1 — Table S1. Specific primers. Specific primers used in this study. (DOC 29 kb) [file 13046_2018_882_MOESM1_ESM.doc]

Specific primers are as follows:

| CCND2(human)-F | ttacctggaccgtttcttgg |
| --- | --- |
| CCND2(human)-R | gaggcttgatggagttgtcg |
| [EML4 (human)-F](http://batchprimer3.bioinformatics.ucdavis.edu/batch_primers/60.172.183.175_1504167635/60.172.183.175_15041676352.html) | cgctgtatggaagggtgtg |
| [EML4 (human)-R](http://batchprimer3.bioinformatics.ucdavis.edu/batch_primers/60.172.183.175_1504167635/60.172.183.175_15041676352.html) | tgggttgcttggaatctctc |
| [FGF9 (human)-F](http://batchprimer3.bioinformatics.ucdavis.edu/batch_primers/60.172.183.175_1504167635/60.172.183.175_15041676353.html) | ggactaaacggcaccagaaa |
| [FGF9 (human)-R](http://batchprimer3.bioinformatics.ucdavis.edu/batch_primers/60.172.183.175_1504167635/60.172.183.175_15041676353.html) | ccatccaagcctccatcata |
| [GADD45G (human)-F](http://batchprimer3.bioinformatics.ucdavis.edu/batch_primers/60.172.183.175_1504167635/60.172.183.175_15041676354.html) | tgatcgcactatgactctgga |
| [GADD45G (human)-R](http://batchprimer3.bioinformatics.ucdavis.edu/batch_primers/60.172.183.175_1504167635/60.172.183.175_15041676354.html) | ccagcacacagaaggtcaca |
| [KRAS (human)-F](http://batchprimer3.bioinformatics.ucdavis.edu/batch_primers/60.172.183.175_1504167635/60.172.183.175_15041676355.html) | catcagcaaagacaagacagaga |
| [KRAS (human)-R](http://batchprimer3.bioinformatics.ucdavis.edu/batch_primers/60.172.183.175_1504167635/60.172.183.175_15041676355.html) | ggcatcatcaacacccagat |
| [MAT2A (human)-F](http://batchprimer3.bioinformatics.ucdavis.edu/batch_primers/60.172.183.175_1504167635/60.172.183.175_15041676356.html) | tcccatcagagtccacacaa |
| [MAT2A (human)-R](http://batchprimer3.bioinformatics.ucdavis.edu/batch_primers/60.172.183.175_1504167635/60.172.183.175_15041676356.html) | gtccagtcaaaccagcatca |
| [MAP3K12 (human)-F](http://batchprimer3.bioinformatics.ucdavis.edu/batch_primers/60.172.183.175_1504167635/60.172.183.175_15041676357.html) | ggcaaagcctactccactga |
| [MAP3K12 (human)-R](http://batchprimer3.bioinformatics.ucdavis.edu/batch_primers/60.172.183.175_1504167635/60.172.183.175_15041676357.html) | gtctcgcaccttcttcacag |
| PIK3R1 (human)-F | gccactttggatttcatcag |
| PIK3R1 (human)-R | caatgctcccacttctacgc |
| [TGFBR1(human)-F](http://batchprimer3.bioinformatics.ucdavis.edu/batch_primers/60.172.183.175_1504167635/60.172.183.175_15041676358.html) | gttccgtgaggcagagattt |
| [TGFBR1(human)-R](http://batchprimer3.bioinformatics.ucdavis.edu/batch_primers/60.172.183.175_1504167635/60.172.183.175_15041676358.html) | ccgtggacagagcaagttt |
| [SMAD9(human)-F](http://batchprimer3.bioinformatics.ucdavis.edu/batch_primers/60.172.183.175_1504167635/60.172.183.175_150416763511.html) | gagaggtgtatgccgagtgc |
| [SMAD9(human)-R](http://batchprimer3.bioinformatics.ucdavis.edu/batch_primers/60.172.183.175_1504167635/60.172.183.175_150416763511.html) | aaagccgtggtgaactgact |
| WNT8B (human)-F | tttgcccactgtctgctacc |
| WNT8B(human)-R | cctcttccatcttcaacctctg |
| WNT10A (human)-F | cgtgctcaatgccaacac |
| WNT10A(human)-R | cgctctctcggaaacctctg |
