# Supplementary figures and images for: miR-4317 suppresses non-small cell lung cancer (NSCLC) by targeting fibroblast growth factor 9 (FGF9) and cyclin D2 (CCND2)
Source: J Exp Clin Cancer Res. 2018 Sep 18;37:230. doi: 10.1186/s13046-018-0882-4 (PMC6145328; doi:10.1186/s13046-018-0882-4)

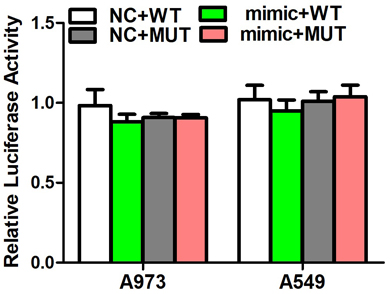

Supplement: Supplementary file 2 — Figure S1. miR-4317 overexpression inhibited cell proliferation, colony formation, and migration. a Quantitation of the miR-4317 level after transfection of miR-4317 mimic in A549 cell lines. b The cell growth curve was measured by MTS after transfection of miR-4317 mimic in A549 cell lines, and the OD570 was normalized to the start point (0 h). c Representative images and quantitation of colony formation after transfection of miR-4317 mimic in A549 cell lines. d Representative images and quantitation of the transwell assay after transfection of miR-4317 mimic in A549 cell lines. e miR-4317 induced cell cycle arrest at G1/S phase. Data are presented as the mean values ± SD from triplicate experiments. ♦p < 0.05. (JPG 86 kb) [file 13046_2018_882_MOESM2_ESM.jpg]

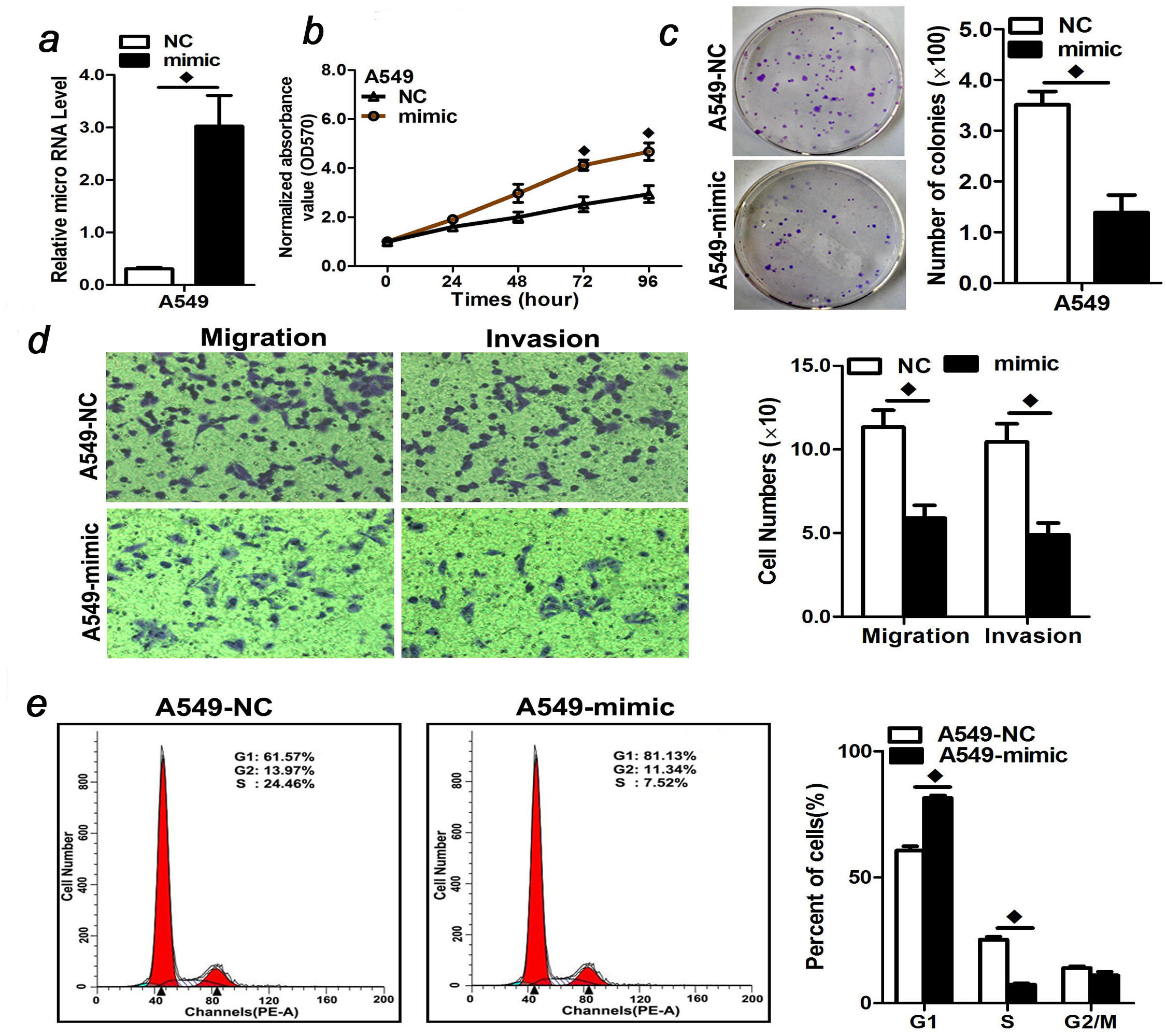

Supplement: Supplementary file 3 — Figure S2. Repression of miR-4317 expression significantly promoted cell growth, colony formation, and migration in H157 cells. a Quantitation of the miR-4317 level after transfection of miR-4317 inhibitor in H157 cell lines. b The cell growth curve was measured by MTS after transfection of miR-4317 inhibitor in H157 cell lines, and the OD570 was normalized to the start point (0 h). c Representative images and quantitation of colony formation after transfection of miR-4317 inhibitor in H157 cell lines. d Representative images and quantitation of the transwell assay after transfection of miR-4317 inhibitor in H157 cell lines. e miR-4317 induced cell cycle arrest at G1/S phase. Data are presented as the mean values ± SD from triplicate experiments. ♦p < 0.05. (JPG 590 kb) [file 13046_2018_882_MOESM3_ESM.jpg]

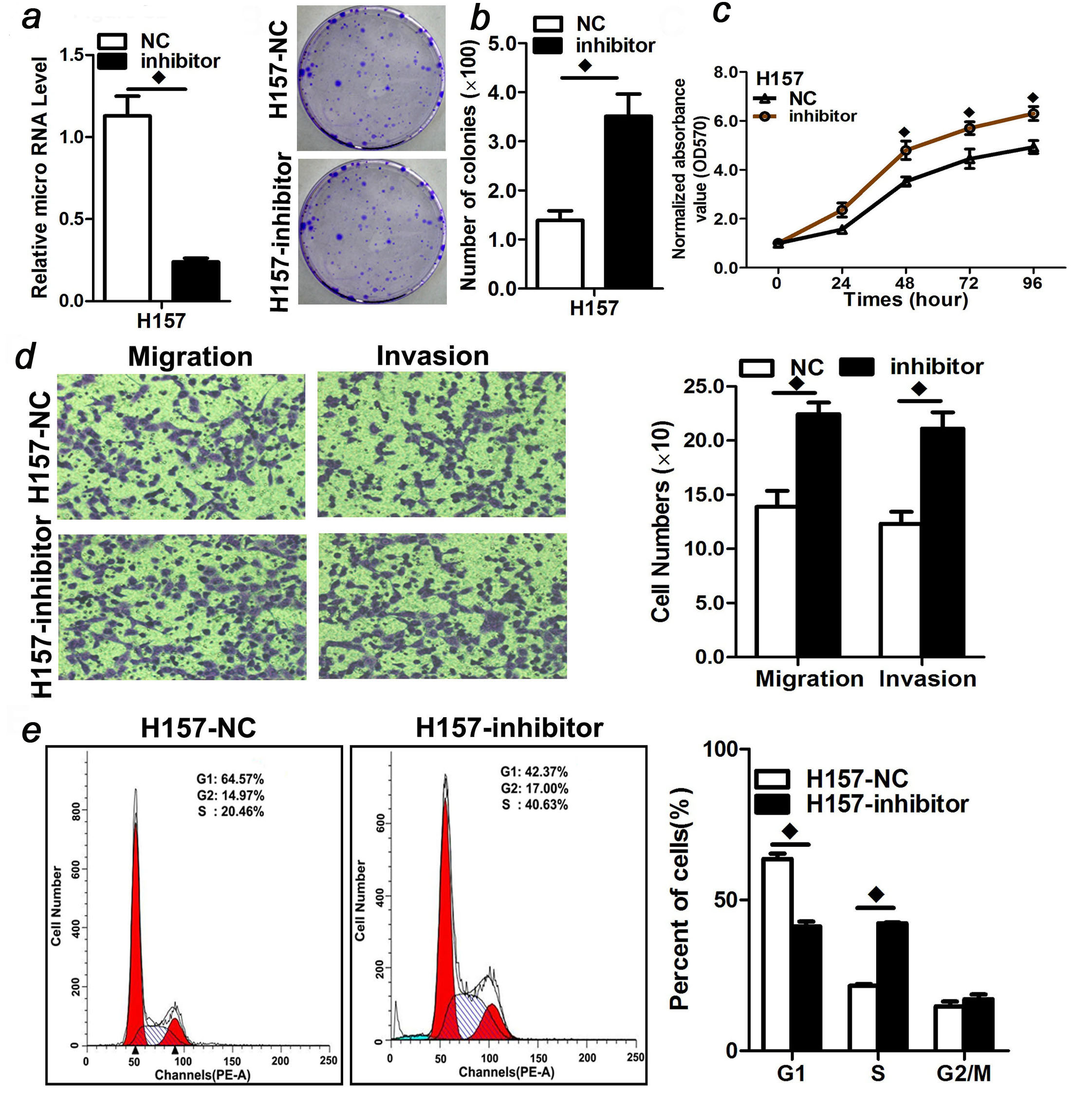

Supplement: Supplementary file 4 — Figure S3. Dual-luciferase reporter assay. The relative luciferase activity was normalized to the Renilla luciferase activity assay after co-transfection of cells with miR-4317 mimic and pmiR-RB-REPORT™ constructs containing WT or MUT TGFBR1 3’-UTR in A973 and A549 cell lines. (JPG 639 kb) [file 13046_2018_882_MOESM4_ESM.jpg]
